# Isolation of deer tick virus (Powassan virus, lineage II) from *Ixodes scapularis* and detection of antibody in vertebrate hosts sampled in the Hudson Valley, New York State

**DOI:** 10.1186/1756-3305-6-185

**Published:** 2013-07-15

**Authors:** Alan P Dupuis II, Ryan J Peters, Melissa A Prusinski, Richard C Falco, Richard S Ostfeld, Laura D Kramer

**Affiliations:** 1 The Arbovirus Laboratories, Wadsworth Center, New York State Department of Health, 5668 State Farm Rd, Slingerlands, NY, 12159, USA; 2 Vector Ecology Laboratory, Bureau of Communicable Disease Control, New York State Department of Health, Albany, NY, 12237, USA; 3 Vector Ecology Laboratory, New York State Department of Health, Louis Calder Center, Fordham University, Armonk, NY, 10504, USA; 4 Cary Institute of Ecosystem Studies, 2801 Sharon Turnpike, Millbrook, NY, 12545, USA; 5 Department of Biomedical Sciences, School of Public Health, University at Albany, State University of New York, 1400 Western Ave, Albany, NY, 12222, USA

**Keywords:** Powassan Virus, Deer Tick Virus, *Ixodes Scapularis*, Ticks, Arbovirus, Flavivirus, Mammals, Birds, Serosurvey, Antibodies

## Abstract

**Background:**

Deer tick virus, DTV, is a genetically and ecologically distinct lineage of Powassan virus (POWV) also known as lineage II POWV. Human incidence of POW encephalitis has increased in the last 15 years potentially due to the emergence of DTV, particularly in the Hudson Valley of New York State. We initiated an extensive sampling campaign to determine whether POWV was extant throughout the Hudson Valley in tick vectors and/or vertebrate hosts.

**Methods:**

More than 13,000 ticks were collected from hosts or vegetation and tested for the presence of DTV using molecular and virus isolation techniques. Vertebrate hosts of *Ixodes scapularis* (black-legged tick) were trapped (mammals) or netted (birds) and blood samples analyzed for the presence of neutralizing antibodies to POWV. Maximum likelihood estimates (MLE) were calculated to determine infection rates in ticks at each study site.

**Results:**

Evidence of DTV was identified each year from 2007 to 2012, in nymphal and adult *I. scapularis* collected from the Hudson Valley. 58 tick pools were positive for virus and/or RNA. Infection rates were higher in adult ticks collected from areas east of the Hudson River. MLE limits ranged from 0.2-6.0 infected adults per 100 at sites where DTV was detected. Virginia opossums, striped skunks and raccoons were the source of infected nymphal ticks collected as replete larvae. Serologic evidence of POWV infection was detected in woodchucks (4/6), an opossum (1/6), and birds (4/727). Lineage I, prototype POWV, was not detected.

**Conclusions:**

These data demonstrate widespread enzootic transmission of DTV throughout the Hudson Valley, in particular areas east of the river. High infection rates were detected in counties where recent POW encephalitis cases have been identified, supporting the hypothesis that lineage II POWV, DTV, is responsible for these human infections.

## Background

*Powassan virus* (POWV; family *Flaviviridae*, genus *Flavivirus)* is a member of the mammalian tick-borne encephalitis virus group [1,2]. POWV was first isolated and identified from brain tissue of a fatal case of encephalitis in 1958 in Powassan, Ontario, Canada [3]. POWV is composed of two lineages, lineage I (prototype POWV) and lineage II (*deer tick virus*; DTV), with distinct transmission cycles [4-6]. With the exception of a few human isolates, the majority of lineage I strains isolated in N. America have been primarily from *I. cookei* ticks and their hosts, woodchucks (*Marmota monax*), mustelids, and wild canids [7-10]. Lineage II strains have been isolated predominantly from *I. scapularis* ticks and/or white-footed mice (*Peromyscus leucopus*) [6,11-13]. A virus isolated in 1952 from *Dermacentor andersoni* ticks collected in Colorado [14], and a virus isolated from the brain of a fox in West Virginia, 1977 [5,10] have been subsequently characterized as lineage II strains. DTV is considered a genotype of POWV due to antigenic and genetic similarity [15]. Evidence of POWV transmission has been detected throughout the United States, Canada and the Primorsky krai region of Russia [10,16,17]. Serologic evidence suggests transmission in Mexico as well [18].

Foci of DTV transmission have been detected in Massachusetts, Connecticut, Wisconsin and Minnesota [6,11-13,19,20]. In addition to these foci and the historical presence in Colorado and West Virginia, RNA and/or infectious virus has been isolated from ticks and humans in New York State (NYS) and from a human encephalitis case in Ontario, Canada [5,21-23]. The spirochete responsible for Lyme borreliosis is hyperendemic along the Hudson River Valley, especially the counties east of the river, indicative of the high population levels of its tick host, *I. scapularis*[24-27]. Human incidence of POWV encephalitis has increased in the US and in particular, southeastern NYS [23,28]. At least 10 of 14 seropositive individuals detected during routine clinical testing in NYS reside in Westchester, Putnam, or Dutchess Counties (unpublished data). Two additional seropositive individuals were identified in Albany and Suffolk Counties, locations with burgeoning populations of *I. scapularis*. Two fatal cases of POW encephalitis following infection with lineage II DTV [21,23], were residents of Putnam County. To assess the presence and/or distribution of POWV, in particular DTV, in the tick and vertebrate host communities and determine if both lineages of POWV occur in this region, we took advantage of NYS Department of Health (NYSDOH) tick-borne pathogen surveillance activities throughout the mid- and lower Hudson Valley, we utilized a well characterized *I. scapularis*/*Borrelia burgdorferi* study site, Cary Institute of Ecosystem Studies (CIES), Dutchess County, and we conducted intensified sampling at an established NYSDOH tick surveillance site in Putnam County, the location of the earliest isolate of DTV in NY.

## Methods

### Field sites

**Figure 1:**
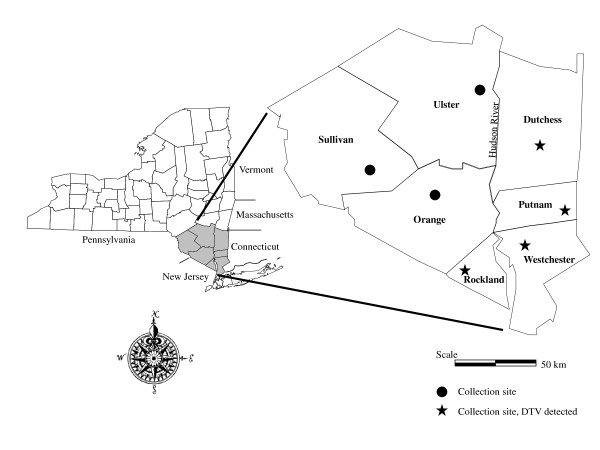
**Map of collection sites in the Hudson Valley, New York State.** Shaded counties and collection sites are shown in projection.

NYSDOH tick-borne pathogen surveillance includes sampling host seeking ticks at sites throughout the Hudson River Valley. Nymphal and adult ticks were collected from Dutchess, Putnam, and Westchester Counties on the east side of the river and from Sullivan, Ulster, Orange and Rockland Counties on the west side (Figure 1). More intensive sampling of questing and replete ticks, mammals, and birds was conducted at a long term study site in Dutchess County (CIES). Questing ticks and birds were also intensively sampled at the surveillance site in Putnam County.

### Tick collections

Ticks were collected by a variety of methods. Questing *I. scapularis* nymphal and adult ticks were collected by standard drag-sampling protocols [25]. A 1 m × 1 m white corduroy cloth was dragged along the ground and flagged across low brush and vegetation. Questing *I. cookei* were collected near animal burrows using the drag cloth and in the burrows using a 20 ft plumber’s snake with white faux fur or corduroy material secured at the end by zip ties. Replete larval and nymphal *I. scapularis* and *I. cookei* were collected from trapped mammals and birds following field and animal husbandry methods of LoGiudice and colleagues [29,30]. Ticks that dropped off their vertebrate hosts were collected, quickly surface sterilized by rinsing with a 10% bleach solution and allowed to molt. Newly molted nymphs were sent to the Arbovirus Laboratories, Wadsworth Center, NYSDOH, for testing. Questing ticks were sorted by species and developmental stage and placed in glass vials containing moistened Plaster of Paris™ until processing or immediately frozen once pooled. For ticks that were maintained alive until processing, glass vials were kept at 20-25° C and monitored for contamination. Ticks were sorted into pools of 1–10 adults or up to 25 nymphs, by site, species, developmental stage, and sent to the Arbovirus Laboratories for testing. Questing tick collections began in fall, 2007 and continued April-November thereafter through spring, 2012. Tick collections were not standardized and coincided with field support availability.

### Vertebrate host collections

#### Mammals

Mammal collection and husbandry methods were approved by CIES IACUC (#09-01I). Coincident with peak larval activity (August), small mammals (mouse-to-chipmunk size)were collected in Sherman live-traps arranged in 8 × 8 array grids, with 15 m spacing between trap stations, covering 1.1 ha. In addition to the 64 Sherman traps per grid, 8 medium sized (15 × 15 × 48 cm) Tomahawk live traps, for catching squirrel-sized animals were set at every other grid point. Larger mammals (raccoons, opossums and skunks) were sampled using large (25 × 30 × 81 cm) Tomahawk live traps placed opportunistically. All traps were covered with plywood boards for protection from sun and rain. Traps were set between 1600 and 1800 h and checked the following morning between 0800 and 1200 h. Because shrews (*Sorex cinereus* and *Blarina brevicauda*) have poor capture probability and survival in Sherman traps, dry pitfall trap arrays, monitored every three hours were used to collect these species. Like the larger Tomahawk traps, these traps were placed opportunistically. Animals were kept in their traps, supplied with bait and apple slices for food and moisture, and driven immediately to the CIES Rearing Facility for temporary holding. Animals were placed in appropriately-sized cages made of ¼ inch mesh galvanized hardware cloth, and supplied with food and water *ad lib*.

Blood samples were collected from species approved by CIES IACUC 09-03I. Blood, 0.05-0.1 mL, was collected from the submandibular vein of *P. leucopus* and from the retro-orbital sinus or from the lateral or medial saphenous veins of larger mammals following inhalant anesthesia (mice, chipmunks: a 20–30% v/v mixture of isoflurane and propylene glycol) or injectable anesthesia (larger mammals: intramuscular Ketamine (70-90 mg/kg) and Xylazine (10 mg/kg)). After 3–5 days in captivity all mammals were released at the point of capture.

#### Birds

Avian collections were conducted at one site each in Dutchess (CIES) and Putnam Counties. Collections took place 2 days/month at each site (March-October, 2011). Blanket mist netting was used to sample the greatest number of passerine and near passerine birds. All captured birds were sexed and aged if possible [31], and approximately 0.075 mL blood was collected in microhematocrit tubes by lancing the ulnar vein of the right wing with a 27 g needle. The blood was expelled into cryotubes containing 0.675 mL of BA-1 diluent consisting of M199 medium with Hank’s salts, 1% bovine albumin, TRIS base (tris [hydroxymethyl] aminomethane), sodium bicarbonate, 20% fetal bovine serum (FBS), and antibiotics. Birds were marked by clipping a retrix or secondary feather denoting month of capture. Birds were examined for ticks, and any attached ticks located around the head of the bird were removed using forceps, placed in snap cap vials containing moistened Plaster of Paris™, and held at room temperature temporarily until processing. Separate vials were used for each bird. At CIES, birds of target species were temporarily housed for the collection of replete larvae. After molting, nymphs were then submitted to the Arbovirus Laboratories for testing. Bird sampling and housing was conducted under CIES IACUC (09-01I) and Wadsworth Center IACUC (09–412), US Fish & Wildlife Migratory Bird Scientific Collecting Permit (MB035731), and NYS Department of Environmental Conservation Permit (LCP: Scientific #1236).

### Virus isolation

All tick samples regardless of species were processed for virus isolation. Individual or pooled ticks were frozen at −80°C immediately prior to homogenization. Ticks were placed in snap capped tubes containing a 5 mm stainless steel BB and 1.0 mL mosquito diluent (PBS supplemented with 20% heat-inactivated FBS, 100 units/ml of penicillin, 100 μg/ml of streptomycin, 10 μg/ml Gentamycin, 1 μg/ml Fungizone). Ticks were homogenized using a Retsch Mixer Mill, MM 301 (Retsch Inc., Newtown, PA) at 24 cycles/second for two 2 minute cycles. Tubes were then centrifuged at 16,100 × g for 2 minutes in a refrigerated Eppendorf 5415 R microfuge. 0.1 mL of supernatant was inoculated onto confluent monolayers of baby hamster kidney cells, (BHK-21), in 6 well tissue culture plates, maintained at 37°C for 7–8 days and examined daily for cytopathic effect (CPE). A 0.1 mL aliquot from any culture exhibiting CPE was passed to fresh monolayers and the remainder of the sample was harvested for an isolate and confirmation of POWV presence, regardless of lineage, by qRT-PCR. Diluted vertebrate blood was centrifuged for 5 minutes. 0.1 mL of supernatant was added to BHK-21 and African green monkey kidney (Vero) cell cultures (ATCC, Manassas, VA) and monitored as above.

### Serology

All vertebrate blood samples were screened for POWV neutralizing antibody utilizing a plaque reduction neutralization test (PRNT) and 80% cut-off at serum dilutions of 1:10. Endpoint titrations of reactive sera, utilizing a 90% cutoff (PRNT90) were then performed as described [32] against prototype POWV (strain LB). Prototype POWV was selected for use in the PRNT due to cross-reactivity with DTV [15]. PRNT90 positive sera were screened against West Nile virus (WNV) and St. Louis encephalitis virus (SLEV) to rule out infection with another member of the Flavivirus genus.

### RT-PCR

Ticks collected prior to spring, 2009 and all CPE positive cell cultures were screened by real time RT-PCR (TaqMan) targeting the NS5 region of the POWV genome. RNA was extracted according to manufacturer protocols using the QIAmp Viral RNA mini kit (Qiagen Inc., Valencia, CA). RT-PCR assays were performed on ABI Prism 7000 or 7500 Sequence Detectors using TaqMan One-Step RT-PCR master mix, formerly Applied Biosystems (Life Technologies, Grand Island, NY). Probes contain a 5′-reporter FAM (6-carboxyfluorescein) and a 3′-quencher TAMRA (6-carboxy-N,N,N,N-tetramethylrhodamine). One primer and probe set was used for the detection of Lineage I and II POWV (FWD- CATAGCRAAGGTGAGATCCAA; REV- CTTTCGAGCTCCAYTTRTT; probe- AGCTCTGGGCGCATGGTYGGATGAACA). A second primer and probe set was used for confirmation of DTV isolates (FWD- GATCATGAGAGCGGTGAGTGACT; REV- GGATCTCACCTTTGCTATGAATTCA; Probe- TGAGCACCTTCACAGCCGAGCCAG). Reaction mixtures were set at thermal cycling conditions of 48°C for 30 mins, 95°C for 10 mins, and 40 cycles of 95°C for 15 sec and 60°C for 1 min.

### Statistics

Maximum Likelihood Estimates (MLE) per 100 ticks with 95% confidence intervals were reported for adult ticks for each county and region wide.

## Results

Between fall, 2007 and spring, 2012 more than 13,500 nymphal and adult ticks of seven species (*I. scapularis, I. cookei, I. dentatus, I. marxi, I. texanus, Dermacentor variablis, Haemaphysalis leporispalustris)* were collected. Collections of *I. cookei*, *I. dentatus, I. marxi, I. texanus, D. variablis* and *H. leporispalustris* accounted for only 1.4% of the total number of ticks sampled. Over 6,100 (3,888 nymphs, 2,231 adults) questing *I. scapularis* were collected. Of the adults, 1,153 were females and 1,078 were males. Prior to spring 2009 all questing tick samples (2,840 nymphs, 145 adults) and nymphs (N = 2,355) that were originally collected as replete larvae from known hosts in 2009 were tested by RT-PCR. Positives were tested by virus isolation. Beginning in spring 2009, all questing ticks (1,048 nymphs, 2,086 adults) and more than 4,700 ticks collected from vertebrate hosts after 2009 were tested by virus isolation and isolates were confirmed by RT-PCR. DTV RNA was detected in 10 pools (9 nymphal, 1 adult) of questing *I. scapularis* collected in fall, 2007 and spring, 2008 from sites in three counties. Positive nymphs were collected in Dutchess and Westchester Counties. The positive adult pool was collected in Putnam County. DTV was isolated from six of the 10 RNA positive pools. No virus was isolated from more than 1,000 questing nymphs collected and tested after spring, 2009.

Additionally, virus was isolated from 43 of 870 pools (4.9%) of questing adults collected at sites in four counties (Putnam 27, Dutchess 9, Westchester 5, Rockland 2) during the period fall, 2009 through spring, 2012(Table 1). DTV was not detected in 335 adult *I. scapularis* collected in Orange, Sullivan, and Ulster Counties, counties north and west of Rockland. Virus was detected in 22 male tick pools and 21 female pools. The infection rate for adult ticks in the counties where DTV was identified was 2.30 per 100 (lower limit 1.69-3.05 upper limit, 95% CI).

**Table 1:** **Infection rates of questing adult *I. scapularis, *collected in 7 Lower Hudson Valley Counties, NY 2009-2012**

**County**	**No. ticks (Total Pools) Male/Female**	**No. DTV isolates Male/Female**	**Infection rate (95% CI)**^**a**^
Dutchess	421/462 (392)	5/4	1.05 (0.52-1.91)
Putnam	302/371 (406)	16/11	3.84 (2.60-5.46)
Westchester	118/84 (21)	0/5	2.73 (1.03-6.04)
Rockland	67/71 (15)	0/2	1.45 (0.27-4.66)
Orange	74/73 (16)	0/0	0.0 (0.00-2.30)
Ulster	46/37 (9)	0/0	0.0 (0.00-3.77)
Sullivan	50/55 (11)	0/0	0.0 (0.00-3.01)

^a^Infection rate expressed as Maximum Likelihood Estimate/100 ticks. The ranges in parentheses are the 95% confidence intervals representing upper and lower limits.

Recently molted nymphal, *I. scapularis* (N = 5,251) and *I. cookei* (N = 99), that fed as larvae from trapped/netted vertebrate hosts (N = 250), were tested. DTV RNA was detected in five pools, all *I. scapularis*. Animals that harbored the positive larvae were two Virginia opossums (*Didelphis virginiana*), two striped skunks (*Mephitis mephitis*) and one raccoon (*Procyon lotor*). These animals were trapped in 2009. 848 ticks collected from white-footed mice were negative for virus and RNA. Infectious virus was not isolated from DTV RNA positive nymphal pools and was not isolated from the blood of any mammalian host sampled (N = 64) (Table 2).

**Table 2:** ***I. scapularis *and *I. cookei *nymphs collected as replete larvae from vertebrate hosts trapped/netted at Dutchess County site (CIES), 2008-2011**

**Host species**	**No. individuals**	**No. *I. scapularis *nymphs (# pools)**	**No. *I. cookei *nymphs (# pools)**	**No. DTV positive *I. scapularis *pools**^**a **^**(# indiv. hosts)**	**Seropositivity no. pos/No. bled**^**b**^
Short-tailed shrew	11	108 (48)			
*Blarina brevicauda*					
Virginia opossum	25	1265 (235)		2 (2)	1/6^c^
*Didelphis virginiana*					
Long-tailed weasel	3	0	1 (1)		0/3
*Mustela frenata*					
Woodchuck	8	6 (2)	41 (26)		4/6
*Marmota monax*					
Striped skunk	5	109 (34)	9 (6)	2 (2)	
*Mephitis mephitis*					
White-footed mouse	87	848 (161)			0/49
*Peromyscus leucopus*					
Raccoon	33	1654 (268)	47 (14)	1	
*Procyon lotor*					
Gray squirrel	19	308 (69)			
*Sciurus carolinensis*					
Red squirrel	10	456 (56)			
*Tamiasciurus hudsonicus*					
Eastern chipmunk	14	63 (36)			
*Tamias striatus*					
Eastern cottontail	3	38 (8)	1 (1)		
*Sylvilagus floridanus*					
American robin	13	55 (39)			
*Turdus migratorius*					
Gray catbird	2	32 (7)			
*Dumetella carolinensis*					
Veery	7	150 (34)			
*Catharus fuscescens*					
Woodthrush	10	159 (36)			
*Hylocichla mustelina*					
TOTAL	250	5251 (1033)	99 (48)	5	5/64

^a^Number of DTV RNA positive tick pools. Infectious virus was not recovered.

^b^Not all individual vertebrate hosts were bled.

^c^Virginia opossums bled did not include the individuals from which DTV positive ticks were collected.

Specific neutralizing antibody to POWV was detected in four of six woodchucks (*Marmota monax*), antibody titer range 1:20–1:320, and one of six Virginia opossums, antibody titer 1:80. Antibodies were not detected in three long-tailed weasels (*Mustela frenata*) and 49 white-footed mice.

Blood samples and more than 1,700 partially fed *I. scapularis, I. dentatus, and H. leporispalustris* larvae and nymphs were collected and tested from 727 mist-netted birds representing 57 species at field sites in Dutchess (N = 439 birds) and Putnam (N = 288 birds) counties. At least 96% of ticks identified were *I. scapularis*. All ticks tested were collected from 305 individuals of 32 avian species, though more birds were infested. At least five ticks were collected from the head of 131 individual birds. These individuals accounted for 80% of the ticks collected and tested. Bird species that were observed with particularly heavy tick burdens included the turdids (thrushes, robins), wrens, towhee, mimids (catbird, thrasher), and cardinal (Table 3). No evidence of infectious DTV was detected in partially fed ticks or blood collected from the birds.

**Table 3:** ***I. scapularis *larvae and nymphs collected from mist-netted birds (in taxonomic order), Dutchess and Putnam Counties, 2011**^**a**^

**Host species**	**# Individuals (# Indiv. w/ticks attached)**	**# Ticks sampled (Est. # ticks on individual birds)**	**Avg # ticks sampled/birds sampled (Range)**
American woodcock	3 (2)	5 (5)	1.7 (0–4)
*Scolopax minor*			
Picids (woodpeckers)	8 (0)	0 (0)	0.0
4 species			
Eastern phoebe	17 (0)	0 (0)	0.0
*Sayornis phoebe*			
Other Tyrannids (flycatchers) 4 species	4 (0)	0 (0)	0.0
Red-eyed vireo	9 (1)	1 (3)	0.3 (0–3)
*Vireo olivaceus*			
Blue Jay	4 (4)	25 (45)	11.3 (1–41)
*Cyanocitta cristata*			
Eastern tufted titmouse	15 (4)	15 (17)	1.1 (0–8)
*Baeolophus bicolor*			
Black-capped chickadee	21 (5)	4 (6)	0.3 (0–2)
*Poecile atricapilla*			
Carolina wren	8 (8)	81 (84)	10.5 (3–20)
*Thryothorus ludovicianus*			
House wren	3 (3)	23 (23)	7.7 (1–19)
*Troglodytes aedon*			
American robin	9 (8)	56 (115)	12.8 (0–40)
*Turdus migratorius*			
Wood thrush	24 (22)	90 (142)	5.9 (0–18)
*Hylocichla mustelina*			
Veery	51 (47)	386 (600)	11.8 (0–40)
*Catharus fuscescens*^b^			
Other Turdids (thrushes)	12 (1)^c^	2 (2)	0.2 (0–2)
3 species			
Gray catbird	215 (157)	392 (671)	3.1 (0–36)
*Dumetella carolinensis*^b^			
Brown thrasher	6 (6)	33 (37)	6.2 (2–15)
*Toxostoma rufum*			
Blue-winged warbler	5 (3)	3 (4)	0.8 (0–2)
*Vermivora cyanoptera*			
Black-and-white warbler	2 (2)	11 (11)	5.5 (3–8)
*Mniotilta varia*			
American redstart	2 (2)	4 (6)	3.0 (2–4)
*Setophaga ruticilla*			
Ovenbird	16 (11)	26 (38)	2.4 (0–11)
*Seiurus aurocapillus*			
Common yellowthroat	8 (3)	16 (18)	2.3 (0–9)
*Geothlypis trichas*			
Other Parulids (warblers)	25 (4)^d^	7 (8)	0.3 (0–4)
11 species			
Northern cardinal	22 (18)	94 (108)	4.9 (0–26)
*Cardinalis cardinalis*^b^			
Rose-breasted grosbeak	6 (3)	0 (6)	1.0 (0–2)
*Pheucticus ludovicianus*			
Indigo bunting	4 (2)	9 (11)	2.8 (0–10)
*Passerina cyanea*			
Eastern towhee	46 (42)	418 (586)	12.7 (0–80)
*Pipilo erythrophthalmus*^b^			
Grasshopper sparrow	7 (6)	9 (12)	1.7 (0–7)
*Ammodrammus savannarum*			
Savannah sparrow	3 (2)	44 (44)	14.7 (0–40)
*Passerculus sandwichensis*			
White-throated sparrow	42 (1)	1 (1)	0.0 (0–1)
*Zonotrichia albicollis*			
Song sparrow	13 (5)	14 (29)	2.2 (0–11)
*Melospiza melodia*			
Other Emberizids (sparrows) 2 species	7 (4)^e^	5 (6)	0.9 (0–4)
Common grackle	3 (3)	15 (16)	5.3 (4–8)
*Quiscalus quiscula*			
American goldfinch	17 (1)	0 (2)	0.1 (0–2)
*Spinus tristis*			
House sparrow	28 (0)	0 (0)	0.0
*Passer domesticus*			
Others 4 species	4 (2)^f^	2 (3)	0.8 (0–2)
Totals	669 (382)	1791 (2659)	4.0 (0–80)

^a^ 58 Birds netted in fall, 2010 were bled only and are not included in the Table.

^b^ 1 individual was seropositive for POWV neutralizing antibodies.

^c^*I. scapularis* (N = 1) removed from 1 of 9 Hermit thrushes (*Catharus guttatus*).

^*d*^*I. scapularis* observed on and/or removed from 1 of 4 Yellow warblers (*Dendroica petechia*) (N = 4), 1 Worm-eating warbler (*Helmitheros vermivora*) (N = 1), 1 of 2 Louisiana waterthrushes (*Seiurus motacilla*) (N = 1, not removed), and 1 Hooded warbler (*Wilsonia citrine*) (N = 2).

^e^*I. scapularis* observed on and/or removed from 2 of 4 Field sparrows (*Spizella pusilla*) (N = 2, 1 not removed), 2 of 3 Unidentified sparrows (N = 5).

^f^*I. scapularis* observed on and/or removed from 1 White-breasted nuthatch (*Sitta carolinensis*) (N = 1, not removed) and 1 Red-winged blackbird (*Agelaius phoeniceus*) (N = 2).

Serologic evidence of POWV infection was detected in 4 of 727 (0.55%) passerine and near passerines mist-netted. Specific neutralizing antibody to POWV was detected in one each of veery (*Catharus fuscescens*), gray catbird (*Dumetella carolinensis*), northern cardinal (*Cardinalis cardinalis*), and Eastern towhee (*Pipilo erythrophthalamus*) using the PRNT90. These four species accounted for approximately 50.0% of the total number of birds sampled and 72.0% of the total number of ticks collected and tested from birds. Of note, all seropositive birds, 1.4% of 288 birds sampled, were netted at the Putnam County field site where there were numerous DTV isolations from questing ticks. None of these birds were recaptures from a previous sampling event. PRNT90 titers were 1:80 for the veery and 1:20 for the catbird, cardinal, and towhee. WNV and SLEV neutralizing antibodies were not detected in the four seropositive birds. WNV was isolated from two hatch year house sparrows netted in Putnam County in late August, 2011.

## Discussion

The detection of DTV in 53 pools (49 infectious virus isolated, 4 RNA positive only) of questing *I. scapularis* collected throughout the Hudson Valley is notable and surpasses the highest total of DTV isolations reported to date [12]. Virus was not isolated from other tick species. Prototype POWV was not detected during the course of this study; however, the number of *I. cookei* ticks tested was >100 fold fewer than the number of *I. scapularis* tested. DTV MLE lower and upper limits indicated between 0.2 and 6.0 DTV infected adult ticks per 100, across sites. Due to differences in collection efforts at our study sites we are unable to provide more robust statistical analyses to assess potential significant spatial and temporal variations at these locations. Furthermore, relative tick abundance across sites was not measured and vertebrate host censuses were not conducted, making it impossible to determine the ecological significance of our results and is beyond the scope of this work.

The observed MLEs are likely highly conservative given the fact that we employed a suboptimal protocol for homogenization of pooled ticks using a mixer mill and stainless steel BBs (versus macerating individual ticks), and we opted for cell culture in 6 and 12 well formats versus RT-PCR assay, in order to detect infectious virus only. These protocols were selected for efficiency, given the large sample sizes, but sacrificed sensitivity. This protocol may have had an impact on nymphal infection rate results. Incomplete homogenization was noted especially for nymphal pools, and prior to the switch from molecular methodologies to virus isolation, DTV RNA was detected in five pools of nymphal ticks originally collected as replete larvae from trapped mammalian hosts. Infectious virus was not isolated from the RNA positive pools, however, the homogenates went through multiple freeze thaw cycles potentially affecting virus viability.

It is not known how the nymphs collected as replete larvae acquired DTV RNA. Three mechanisms are potentially responsible: horizontal transmission from vertebrate host to tick, co-feeding transmission, and transovarial transmission (TOT). Unfortunately these hosts were not bled during captivity to assess viremia. In experimental infection studies, striped skunks developed a trace viremia for one day and opossums developed trace viremias for eight to 11 days following subcutaneous inoculation with >10^3^ LD_50_ POWV [33]. Raccoons have not been assessed for host competence. Though experiments are lacking for co-feeding transmission of POWV, studies involving other members of the tick-borne encephalitis virus group and *I. ricinus* ticks have demonstrated co-feeding or non-viremic transmission [34-36]. This mode of transmission has been hypothesized as being sufficient to maintain the virus in nature [37]. TOT has been documented experimentally for *I. scapularis* and POWV. Larvae from one of six females exposed as nymphs were able to infect a hamster resulting in HI antibody titers, >5,120, at 42 days post tick drop off [38]. In our study, larvae were not tested with the exception of two clutches from *I. cookei* that fed on a trapped woodchuck, and assessment of TOT was beyond the focus of this study.

Virus was not isolated from any of the vertebrate hosts sampled or any partially fed ticks removed from hosts. This is not surprising considering viremia is short-lived in many of the vertebrate hosts sampled [10]. Serologic evidence of POWV exposure was observed in woodchucks, an opossum, and four birds. It is impossible to determine the POWV lineage responsible for infection given the antigenic relatedness of the viruses. It is worth noting that no evidence of POWV/DTV was detected in any derivative (ticks, blood) collected from the white-footed mice sampled in this study, considering they have been implicated as an important host for DTV [19]. This is most likely a function of a limited sample size. Woodchucks have been implicated as an important host for prototype POWV, and the majority of the *I. cookei* ticks tested during this study were collected from woodchucks. *I. cookei* are known to infest opossums as well [39], but both species are also infested with *I. scapularis*. *I. cookei* are more restricted to mammal burrows than are *I. scapularis* and therefore unlikely to feed on birds. *I. scapularis* larvae and nymphs were recorded on a majority of the individual netted birds. Given the number of DTV isolates and MLE at the sampling site, it is likely the resulting antibody was due to DTV infection. Determination of infecting agent is unimportant from a human health standpoint as both POWV lineages are capable of producing severe disease in humans, though acaricidal strategies could target the most likely vector.

The role of birds in POWV transmission has yet to be determined. Birds have been implicated as important hosts for other tick-borne flaviviruses, including Louping Ill virus [40] and TBEV [41]. Reports from the former Soviet Union suggest POWV has been isolated from numerous species of birds, in particular Anseriforms (ducks) [10]. HI antibodies have been documented in a number of birds sampled in the USA, Canada, and the former Soviet Union [10,42,43]. This assay is not specific for POWV, and neutralizing antibody was confirmed in only two of the birds in a single study [43]. Regardless of a direct role in virus amplification, birds have the potential to transport infected ticks, as evidenced with *B. burgdorferi* and *I. auritulus* and *I. scapularis* ticks [44,45] and are the bloodmeal source of immature stages. Furthermore, a competent or serologically naïve vertebrate host is not necessarily required for co-feeding transmission [36].

The results from this field investigation have provided a foundation and guidance for future studies of the ecology of DTV in Southeastern NYS, and may be relevant to other foci in the Midwest and Atlantic coastal regions. It is evident that DTV is widespread in the adult *I. scapularis* population in these areas, and certain tick populations have high infection rates. The apparent increase in clinical POW encephalitis cases, especially in the Hudson Valley, may be the result of improved surveillance and diagnostics since the introduction of WNV. However, it is conceivable that increased human incidence is attributable to the “escape” of POWV from the cryptic *I. cookei* driven transmission cycle where human exposure in NYS is 10–15 times lower than *I. scapularis* (unpublished data), to a transmission cycle facilitated by a competent tick [38] with catholic feeding preferences [29,39], and the ability to transmit virus within 15 minutes of attachment to the host [46].

## Conclusion

Evidence of widespread and continuous (2007–2012) DTV transmission was noted in several counties of the Hudson Valley, NY, concomitant with an apparent increase in the number of diagnosed human POW encephalitis cases since 2004 in the same region. Small to medium sized mammals, such as opossums, woodchucks, and raccoons may be important hosts for amplification of virus or at least tangentially involved in vector maintenance. Specific neutralizing antibodies to POWV were detected in passerines for the first time in the US, supporting earlier findings in British Columbia, Canada [43], indicative of exposure to infected ticks and thus serving as a potential vehicle for dispersal. Results of this study emphasize a need for further investigation to determine risk of human exposure, demarcate the geographic range of DTV transmission in the Hudson Valley and across the range of *I. scapularis*, elucidate important vertebrate hosts, and evaluate/assess the role of alternative transmission cycles (co-feeding, TOT).

## Abbreviations

POWV: Powassan virus; DTV: Deer tick virus; CIES: Cary Institute of Ecosystem Studies; NY(S): New York (State); NYSDOH: New York State Department of Health; PRNT: Plaque reduction neutralization test; MLE: Maximum likelihood estimate; TOT: Transovarial transmission.

## Competing interests

The authors declare that they have no competing interests.

## Authors’ contributions

APD participated in study design, collected samples, performed experiments, analyzed the data, and drafted the manuscript. RJP coordinated sample collections, performed experiments, analyzed data, assisted with manuscript preparation, and provided critical review of manuscript. MAP and RCF coordinated questing tick collections, assisted with manuscript preparation, addition of figure and provided critical review of manuscript. RSO and LDK participated in and coordinated the study’s design and direction, drafted sections of the manuscript and provided critical review of earlier versions. All authors read and approve of the material presented in the manuscript.
